# UAV-Based LiDAR and Multispectral Imaging for Estimating Dry Bean Plant Height, Lodging and Seed Yield

**DOI:** 10.3390/s25113535

**Published:** 2025-06-04

**Authors:** Shubham Subrot Panigrahi, Keshav D. Singh, Parthiba Balasubramanian, Hongquan Wang, Manoj Natarajan, Prabahar Ravichandran

**Affiliations:** Lethbridge Research and Development Center, Agriculture and Agri-Food Canada (AAFC), 5403 1st Avenue South, Lethbridge, AB T1J 4B1, Canada; shubham.panigrahi@agr.gc.ca (S.S.P.); parthiba.balasubramanian@agr.gc.ca (P.B.); hongquan.wang@agr.gc.ca (H.W.); manoj.natarajan@agr.gc.ca (M.N.); prabahar.ravichandran@agr.gc.ca (P.R.)

**Keywords:** dry bean, LiDAR, multispectral, canopy height, crop lodging, seed yield, machine learning

## Abstract

Dry bean, the fourth-largest pulse crop in Canada is increasingly impacted by climate variability, needing efficient methods to support cultivar development. This study investigates the potential of unmanned aerial vehicle (UAV)-based Light Detection and Ranging (LiDAR) and multispectral imaging (MSI) for high-throughput phenotyping of dry bean traits. Image data were collected across two dry bean field trials to assess plant height, lodging and seed yield. Multiple LiDAR-derived features accessing canopy height, crop lodging and digital biomass were evaluated against manual height measurements, visually rated lodging scale and seed yield, respectively. At the same time, three MSI-derived data were used to estimate seed yield. Classification- and regression-based machine learning models were used to estimate key agronomic traits using both LiDAR and MSI-based crop features. The canopy height derived from LiDAR showed a good correlation (R^2^ = 0.86) with measured plant height at the mid-pod filling (R6) stage. Lodging classification was most effective using Gradient Boosting, Random Forest and Logistic Regression, with R8 (physiological maturity stage) canopy height being the dominant predictor. For seed yield prediction, models integrating LiDAR and MSI outperformed individual datasets, with Gradient Boosting Regression Trees yielding the highest accuracy (R^2^ = 0.64, RMSE = 687.2 kg/ha and MAE = 521.6 kg/ha). Normalized Difference Vegetation Index (NDVI) at the R6 stage was identified as the most informative spectral feature. Overall, this study demonstrates the importance of integrating UAV-based LiDAR and MSI for accurate, non-destructive phenotyping in dry bean breeding programs.

## 1. Introduction

High-throughput phenotyping is vital for understanding crop performance, particularly during the increasing demand for sustainable agricultural practices. Traditional methods are often labor-intensive and subjective, limiting their capacity to obtain dynamic plant responses efficiently. In contrast, non-invasive and non-destructive imaging technologies, such as RGB, multispectral, hyperspectral and LiDAR, have provided valuable understandings of plant performance by allowing the rapid collection of large-scale intrinsic phenotypic data [1,2,3,4].

Compared to satellites, unmanned aerial vehicles (UAVs) mounted with imaging systems have become highly desirable in agriculture monitoring [5,6]. The flexibility for altering the imaging parameters such as sensor angle, flight speed and altitude reduces the atmospheric influence on the data collected, making UAVs better than satellites for precision agricultural applications [7,8]. RGB-based imagery has shown good results in estimating canopy height for wheat crops [9]. However, studies have demonstrated that UAVs equipped with LiDAR, multispectral and hyperspectral sensors can effectively monitor various crop traits altogether, contributing to an inclusive understanding of dense and cereal crop structures [10,11]. Moreover, the combination of LiDAR and multispectral imaging has been shown to enhance the accuracy of trait measurements, allowing for better estimation of above-ground biomass and yield for cereal crops and grass [3,12,13]. UAV-derived canopy metrics (e.g., canopy cover and vegetation indices) in pulse crops such as chickpea and dry pea strongly correlated with ground traits, including final seed yield and phenological stages like mid-pod filling and maturity timing. Such image-based features have enabled accurate yield predictions in these pulses using machine learning models (coefficient of determination (R^2^) up to 0.91 in chickpea), highlighting the potential of remote sensing to accelerate legume breeding [14]. In soybean, multi-temporal multispectral imagery coupled with machine learning has been used to monitor growth and forecast yield. Notably, vegetation indices captured at the early pod development stage facilitated early yield prediction with errors as low as 0.5 t/ha [15]. Such LiDAR-derived height metrics can serve as proxies for biomass and lodging risk, traits that are difficult to measure manually at scale.

Lodging resistance is another crucial trait for legumes and pulses that can be evaluated via UAV phenotyping. Lodging (the bending or breaking of stems) can severely reduce yield and crop quality. It has the tendency to raise disease risk (such as white mold) from poor airflow and moisture while slowing harvests. In soybean, yield losses of 18–32% have been recorded when lodging occurs at mid-reproductive stages, and complete lodging by maturity can cut yields by over 30% [16]. Conventional visual scoring of lodging is subjective and inefficient, motivating remote sensing approaches to quantify lodging in breeding plots. These advances illustrate how UAV-based LiDAR and multispectral imagery (MSI), together with machine learning analytics, can enhance the phenotyping of yield-related and stress-adaptive traits in legumes.

Dry bean is an important crop for human nutrition and agricultural economies. UAVs equipped with MSI sensors have reformed data collection in dry bean fields [17]. Recently, Wang et al. [6] showed that a photogrammetrically created digital surface model from RGB and MSI images estimated the dry bean plant height with a lower Pearson correlation coefficient (r) at the mid-flowering stage. The same study observed that the multispectral sensor estimated the seed yield with a lower ‘r’ value at the mid-pod filling (R6) stage. Most recent UAV phenotyping studies in legumes have focused on multispectral or RGB imaging, while the potential of LiDAR in these crops is largely under-explored. Furthermore, the integration of 3D LiDAR data with spectral indices for a more holistic trait estimation has been limited in dry bean breeding trials. This study addresses these gaps by employing a UAV-mounted LiDAR and MSI system to phenotype dry bean field trials, focusing on estimating plant height, crop lodging and seed yield.

The aim of this study was to evaluate the effectiveness of a UAV-based LiDAR and MSI system for characterizing dry bean growth parameters. The specific objectives include: (1) assessing the capability of LiDAR to accurately detect canopy height (CH) (to estimate plant height), crop lodging (CL) and digital biomass (DB) (to estimate seed yield); (2) evaluating the potential of multispectral imaging to estimate seed yield and (3) determining the combined effectiveness of LiDAR and MSI in estimating seed yield for efficient phenotypic trait estimation.

## 2. Materials and Methods

### 2.1. Study Site and Ground Data Collection

The study site is located at an experimental farm of Agriculture and Agri-Food Canada, located at Lethbridge Research and Development Center in Southern Alberta, Canada (49.7077° N and −112.6905° W). In this study, two dry bean field trials, Advanced Yield Trial (AYT) consisting of F_7_ generation lines, including yellow bean (10 entries: YL), pinto bean (13 entries: PT) and great northern bean (20 entries: GN), and a Performance Yield Trial (PeYT) consisting of F_8_–F_10_ generation lines (28 entries) were grown in a randomized-block design. Each entry had four replications. All the data collection was conducted at three dry bean growth stages, i.e., mid-flowering (R1), mid-pod filling (R6) and physiological maturity (R8), and a minimal gap between ground sampling and UAV flights was ensured, as shown in Table 1.

The plant height was measured using rulers for the three stages. In each stage, three data points that are representative of this trait were considered by using the middle two rows of the plot. Lodging resistance was visually rated at R8 maturity stage with a scale of 1 to 5. Seed yield (kg/ha) was determined after harvest.

### 2.2. UAV Image Acquisition

**Figure 1:**
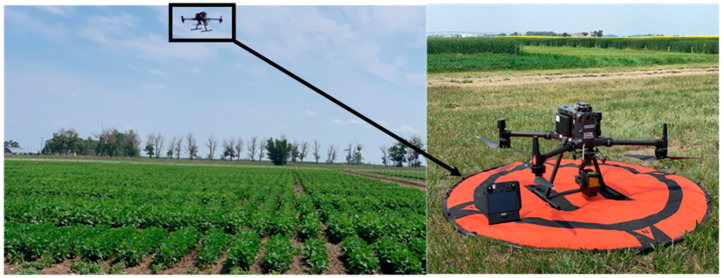
UAV flying over the dry bean plots (**left**) and UAV system with the LiDAR sensor (**right**).

UAV-based LiDAR and MSI data were acquired using the DJI Zenmuse L2 system (SZ DJI Technology Co., Ltd., Shenzhen, China) (20MP, 905 nm; 240,000 pts/s) and the Micasense RedEdge-P sensor (AgEagle Aerial Systems Inc., Wichita, KS, USA) (six bands: Panchromatic, Blue, Green, Red, RedEdge and NIR) at the R1, R6 and R8 growth stages, respectively. Survey planning and camera orientation were managed through the DJI Pilot app (SZ DJI Technology Co., Ltd., Shenzhen, China), with flights executed in a single-grid pattern and the camera positioned at nadir (Figure 1). The UAV operated at a flight altitude of 30 m above ground level with an imaging speed of 3.0 m/s, ensuring 80–85% overlap in both forward and lateral directions, resulting in a ground sampling distance (GSD) of 1.96 cm/pixel.

To enhance positional accuracy, a D-RTK2 Global Navigation Satellite System (GNSS) (SZ DJI Technology Co., Ltd., Shenzhen, China) base station was deployed during image acquisition. All imagery was collected under sunny conditions around solar noon and calibrated using a white reflectance panel and a downwelling irradiance sensor (DLS-2) to correct for variations in lighting. Additionally, four ground control points (GCPs) were placed at each corner of the rectangular field, with coordinates recorded using a multi-frequency GNSS receiver connected to a high-precision base station to improve georeferencing accuracy during post-processing.

### 2.3. Data Processing

#### 2.3.1. LiDAR Point Cloud

**Figure 2:**
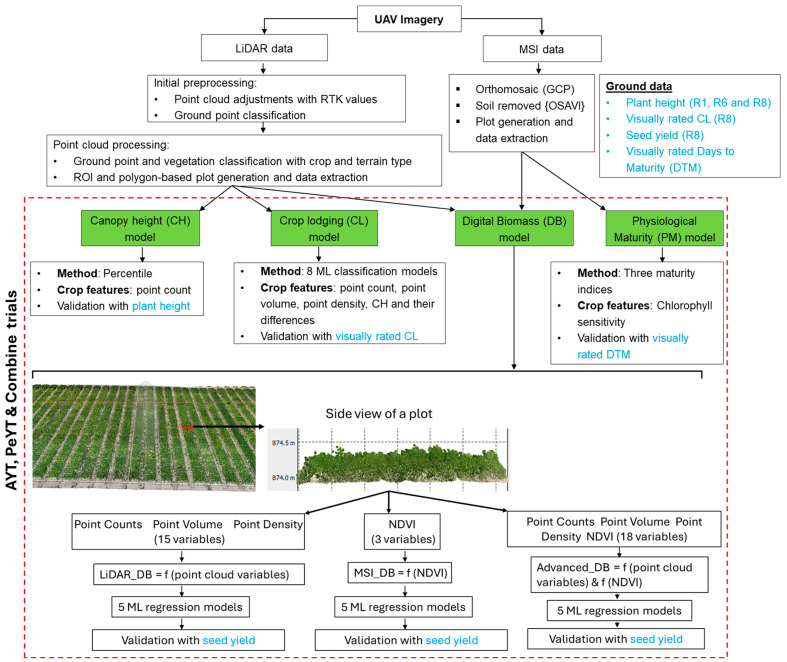
Process flow chart for LiDAR and MSI image data extraction and analysis (ML: Machine learning).

The raw LiDAR image of the R1, R6 and R8 growth stages were imported into DJI Terra v3.8.0 for initial preprocessing (Figure 2), where georeferencing was performed by setting the base station center point using latitude, longitude and real-time kinematic (RTK) altitude data. The process flow chart to generate the processed images and further data extraction and analysis is shown in Figure 2. The processing parameters were configured to achieve 100%-point cloud density, with an effective distance range of 3–300 m in the WGS84 coordinate system. Ground point classification was performed using the flat ground classification method, with a max diagonal distance of 3 m, an iteration angle of 0.3° and an iteration distance of 0.02 m. Accuracy control was implemented by introducing four GCPs, ensuring alignment with the survey reference. The LAS (LiDAR Aerial Survey) file was imported into Agisoft Metashape Professional v2.0.3 (Agisoft LLC, St. Petersburg, Russia) for additional processing, including ground classification and polygon-based plot generation for the field trials.

The region of interest (ROI) was extracted using the crop selection tool, and ground classification was refined by setting the maximum angle and terrain slope at 10° and maximum distance at 0.021 m determined using the point density observed for the three growth stages. Field plots were set out using the 2024 Dry Bean Plots with AYT and PeYT trials, with polygon-based segmentation ensuring accurate spatial referencing. In field trials, plots were drawn using the ‘Draw polygon’ tool. After georeferencing the point cloud, a shapefile of plot polygons (each polygon representing dry bean cultivar plot) was used to clip the point cloud. All points falling inside a given polygon are extracted as that plot’s point cloud.

The final classified LAS file was exported for plot-wise feature extraction and structural trait computation using open-source python (version 3.9) tool. The LAS point cloud file was processed using python code to extract the point-based crop features for all the three stages as shown in Figure 2. A total of 15 LiDAR-derived features were extracted from the point cloud data for each polygon-based plot. These features include vegetation point counts, point density, point volume and CH for R1, R6 and R8 stages, respectively, (making it a total of 12 features) and difference in point density, point volume and CH between R6 and R8 stages (making it a total of 3 features) were also considered for dry bean trait estimation. All the analysis was conducted for AYT, PeYT and a combination of these trials for all the three growth stages.

#### 2.3.2. MSI Data Processing

**Figure 3:**
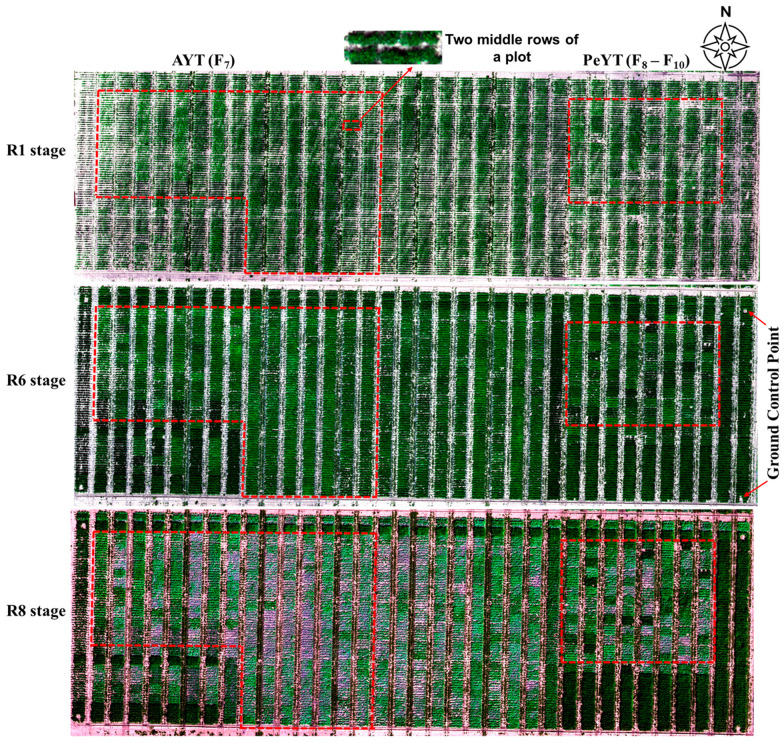
Orthomosaic images for the three dry bean growth stages showing AYT and PeYT trials.

Multispectral images of the R1, R6 and R8 dry bean growth stages were processed in Pix4D Mapper (Prilly, Switzerland) to create orthomosaics (Figure 3). These images were calibrated using the white reflectance panel to compensate for variable light conditions and maintain precision to enable accurate multi-temporal comparisons. The panel’s surface values were provided by MicaSense (AgEagle Aerial Systems Inc., Wichita, KS, USA) to assign a definitive reflectance value during the corrections process. Images of the panel were taken after each flight, and the reflectance calibration was conducted in Pix4D Mapper. Geometric correction was conducted using Pix4D Mapper, where images were co-registered using ground control points (GCPs) to ensure high spatial accuracy.

For plot-wise analysis, the shapefile containing trial labeled plots was overlaid on the orthomosaic in ENVI v6.0 (L3Harris Geospatial Solutions Inc., Boulder, CO, USA) for feature extraction. The Optimized soil-adjusted vegetation index (OSAVI) was used to mask out the background noise, including soil and shadow pixels [18]. Thus, for each plot, only the pixels corresponding to the vegetation were used in the following analysis. The analysis was conducted separately for AYT, PeYT and their combined dataset across all growth stages.

### 2.4. Dry Bean Traits Estimation

The dry bean physiological trait features consisted of CH (to estimate plant height), CL (to estimate lodging) and DB (to estimate seed yield).

#### 2.4.1. Plant Height Estimation

LiDAR imagery data were used to calculate CH based on percentile method to generalize the point-based height distribution within the plots [11]. CH model was used to estimate dry bean plant height. The model was formulated based on the average of top and bottom portions of the dry bean, as shown in Equation (1).

CH = Average of top percentile − Average of bottom percentile(1)

where ‘top’ ranges from 70th to 100th percentile range with an increment of 1, 2 and 5 points and ‘bottom’ ranges from 0 to 10th percentile range with an increment of 1, 2 and 5 points. These increments were selected based on the approach used by ten Harkel et al. [11]. They used a 5-point increment to estimate the percentile ranges for sugar beet crops. As dry bean crops have a denser but lower canopy structure, finer increments such as 1 and 2 points were included alongside 5 points in this study. Based on this formulation, a total of 962 CH model cases were simulated and correlated with average plant height values.

#### 2.4.2. CL Estimation

LiDAR imagery point cloud features were used to estimate visually rated CL. It was determined using a combined classification approach named ‘Low Lodging’ (LL) and ‘High Lodging’ (HL), where the LL category included lodging scales of 1, 2 and 3 while HL included scales of 4 and 5 based on the low data points available for ‘2’, ‘3’ and ‘5’ lodging scales as shown in Table 2. It is to be noted that there were no plots showing lodging scale of 1.

A total of eight ML classification models were explored to train and test original and balanced dataset, respectively, as these models were used for similar legume crops, such as dry peas [19]. The classification models used in this study were Adaptive Boosting (AB), Gradient Boosting (GB), K-Nearest Neighbors (KNN), Light Gradient Boosting Machine (LightGBM), Random Forrest (RF), Support Vector Machine (SVM), Extreme Gradient Boosting (XGBoost) and Logistic Regression (LR). In addition, balanced dataset was obtained using Synthetic Minority Oversampling-Edited Nearest Neighbor (SMOTE-ENN), SMOTE-Tomek Link, Borderline-SMOTE and Adaptive Synthetic (ADASYN) models that were previously explored for dry peas by Bazrafkan et al. [19]. Furthermore, CL estimation was conducted considering R6 and R8 stages to compare the evaluation matrices calculated as shown in Section 2.5 (Equations (10)–(13)) and analyze the feature dominance parameters.

#### 2.4.3. Seed Yield Estimation

LiDAR and MSI imagery data were used to determine dry bean DB, followed by correlating it with actual seed yield (Figure 2). The LiDAR-derived 15 crop features and MSI-derived Normalized Difference Vegetation Index (NDVI) were used to detect the dry bean biomass using MSI (Equation (2)). NDVI was calculated for all three stages using the reflectance values from the near-infrared (NIR) and red spectral bands.

NDVI = (NIR − Red)/(NIR + Red)(2)

where its value ranges from −1 to +1, where higher values indicate dense, healthy vegetation, while lower values suggest sparse or stressed vegetation, bare soil or water bodies. Finally, seed yield estimation was conducted using LiDAR and MSI separately and also by using a combination approach with five different ML regression models such as Artificial Neural Network (ANN), Gradient Boosting Regression Trees (GBRT), Random Forrest (RF), Partial Least Square Regression (PLSR) and Multiple Linear Regression (MLR). These models were selected based on their ability to handle complex, non-linear relationships commonly found in high-throughput phenotyping data. GBRT, in particular, was chosen for its robustness to overfitting, ability to capture intricate feature interactions, and superior performance in a previous study conducted for wheat to estimate biomass [9].

### 2.5. Model Evaluation

For CH model optimization, three evaluation metrics R^2^, root mean square error (RMSE) and mean absolute error (MAE), were determined for 962 cases, and composite scoring was conducted by considering 0.4, 0.4 and 0.2 weightage for each metric, respectively (Equations (3)–(6)). Prior to calculation of the composite score, each matrices were normalized based on the requirements, such as the final score should incorporate high R^2^ and, at the same time, low RMSE and MAE proportions (Equations (7)–(9)). The CH model case with the highest composite score was considered the best model.

Classification models used for CL estimation were evaluated using Equations (10)–(13), and regression models were used to estimate seed yield. The dataset comprised a total of 276 plot-level samples, corresponding to the number of experimental plots across both the AYT and PeYT. All the classification and regression ML models were trained with 80% of the dataset and tested with 20% of the dataset using scikit-learn Python packages. The specific Python function used for this purpose was ‘train_test_split’ from the scikit-learn library. In certain cases, trial-based stratified splitting (AYT vs. PeYT) was performed to evaluate model generalization across independent trials. The input features for the models included 15 LiDAR-derived crop structural traits (e.g., canopy height, point density, point volume and their temporal differences across three stages) and three multispectral features (e.g., NDVI at R1, R6 and R8 stages). The output variables were lodging class (LL or HL) for classification and seed yield for regression. To prevent overfitting and improve generalization, hyperparameter tuning of models such as ANN and GBRT was carried out using 5-fold cross-validation on the training data. For classification models (e.g., for lodging estimation), stratified 5-fold cross-validation was employed to ensure class balance across folds. For regression models, a standard 5-fold cross-validation was applied. Hyperparameter tuning involved varying ANN layer sizes (512 and 256; 1024 and 512; 2048 and 1024 neurons) and GBRT learning rates (0.1; 0.2; 0.3) and selecting the combination yielding the best average training R^2^ across folds. Feature dominance was calculated based on ANOVA and *t*-test for lodging and seed yield estimation. Then, the *p*-values were converted into importance (%) using effect size normalization method [20]. Models were evaluated using classification and regression-based matrices, as shown below:

For regression models:

(3) $$R^{2}=1-\frac{\sum_{i=1}^{n}{\left(x-y\right)}^{2}}{\sum_{i=1}^{n}{\left(x-\bar{x}\right)}^{2}}$$

(4) $$RMSE=\sqrt{\frac{1}{n}\sum_{i=1}^{n}{\left(x-y\right)}^{2}}$$

(5) $$MAE=\frac{1}{n}\sum_{i=1}^{n}\left|x-y\right|$$

(6) $$Composite\;score=0.4\times {normalized\;R}^{2}+0.4\times normalized\;RMSE\;+0.2\times normalized\;MAE$$

(7) $${normalized\;R}^{2}=\frac{{R^{2}}_{i}-min\;R^{2}}{{max\;R}^{2}-min\;R^{2}}$$

(8) $$normalized\;RMSE=\frac{\max\;RMSE-{RMSE}_{i}}{\max\;RMSE-\min\;RMSE}$$

(9) $$normalized\;MAE=\frac{\max\;MAE-{MAE}_{i}}{\max\;MAE-\min\;MAE}$$

where x is actual value; y is predicted value; $\bar{x}$ is mean of actual values; i is current case; n is number of observations; ‘min’ is minimum value of the 962 cases; ‘max’ is maximum value of the 962 cases.

For classification models:

(10) $$Accuracy=\frac{TP+TN}{TP+TN+FP+FN}$$

(11) $$Precision=\frac{TP}{TP+FP}$$

(12) $$Recall=\frac{TP}{TP+FN}$$

(13) $$f1-score=2\times \frac{Precision\times Recall}{Precision+Recall}$$

where TP is true positive; TN is true negative; FP is false positive; FN is false negative.

## 3. Results and Discussion

### 3.1. Plant Height Estimation Using LiDAR

**Figure 4:**
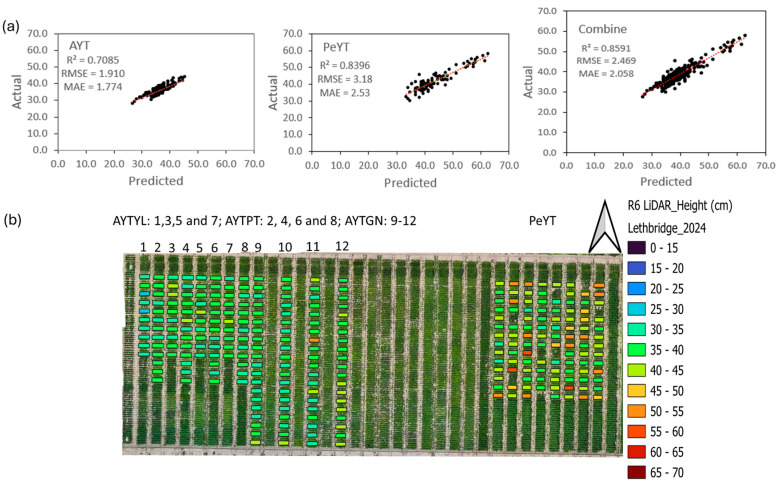
Plant height estimation results for R6 stage (Actual: plant height; predicted: canopy height): (**a**) correlation plots for AYT, PeYT and combine trials; (**b**) estimated CH values for each plot.

The best dry bean CH model obtained for each of the three stages is shown in Equation (14). The canopy height estimation was conducted using the below equation for the R1, R6 and R8 stages. Out of all the three stages, R6 exhibited the highest correlation, as shown in Figure 4. The R1 stage showed an R^2^ of 0.77 for PeYT, 0.76 for AYT and 0.76 for combined trials, and the R8 stage showed an R^2^ of 0.61 for PeYT, 0.63 for AYT and 0.63 for combined trials.

CH = Avg height of top 10 percentile − Avg height of 1st percentile(14)

The R1 stage showed a relatively higher correlation than the R8 stage. Although there was a 5-day delay in obtaining the LiDAR point cloud data for R1 in comparison to only a 1-day delay for the R8 stage, lodging at the R8 stage could have resulted in lower heights. Studies have confirmed that delays in UAV-based data procurements result in lower correlations due to different growth parameters [7,11]. It could also be due to significant differences in canopy height field values observed for the three data points collected across the plots. In the R1 stage, there was a standard deviation of 1 to 15 cm between the three data points, followed by 1 to 11 cm for R8 and 1 to 6 cm for the R6 stage. Possible explanations for the error include the subjective nature of the ground-truth measurements, which were affected by wind or the position of the meter stick. It was also estimated that within each stage, the plots on Range-2 of the AYTYL trial and 21st Pass of AYTGN trials showed the lowest canopy height values in comparison to the rest plots. This was observed mainly due to soil compaction.

Within the trials, PeYT showed a higher correlation than AYT. This was because, within the AYT trail, there were three different sub-trials of Pinto (AYTPT), Great Northern (AYTGN) and Yellow (AYTYL) bean market classes. Each sub-trial likely had different canopy structures, growth patterns or variations in LiDAR reflectance, leading to lower individual correlation values at R6 compared to PeYT. Mainly, AYTGN had different canopy growth in comparison to AYTPT and AYTYL sub-trials, as can be seen in Figure 4.

This suggests that, at the mid-pod filling (R6) stage, structural or canopy characteristics of AYT bean market class types aligned better with LiDAR data compared to PeYT. In soybean, the LiDAR-derived CH model achieved R^2^ of 0.83 at mid-season, outperforming RGB-based height estimation (R^2^ = 0.53) due to photogrammetry’s tendency to smooth canopy variation and underestimated peak heights [21]. Yuan et al. [22] showed similar results for peanut canopy height estimation with an R^2^ of 0.92 using a ground-based LiDAR system. This research utilized a ground-based LiDAR system to assess peanut canopy architecture across different growth stages. The study found that the LiDAR-derived measurements of canopy height and structure had varying correlations with manual field data depending on the growth stage. Notably, at later stages of growth, such as the R6 stage, the LiDAR data provided more accurate representations of the canopy structure, leading to higher correlations with field measurements. Wang et al. [6] showed that a photogrammetrically created digital surface model from an RGB image detected canopy height with ‘r’ of 0.49, while with MSI, the ‘r’ was 0.45 with the ground measurements of AYT plots. However, RGB and MSI sensors showed an ‘r’ of less than 0.32 for PeYT plots. The overall correlation coefficients were low and a severe underestimation was observed in comparison to LiDAR data. This shows the credibility of considering point-based representations of the dry bean crop instead of a 2D averaged raster representation of the top view of the canopy.

### 3.2. CL Resistance Using LiDAR

Among the 8 ML classification models, Gradient Boosting, Random Forest and Logistic Regression showed the best matrices, as shown in Table 3. It can be said that dry bean lodging estimation can be properly conducted using either of the three models because their matrices were not significantly different. These matrices were obtained using the original dataset. Out of all the algorithms used to balance the dataset, the SMOTE-Tomek Link method showed the highest accuracy, precision, recall and F1-score; however, results obtained from the balanced dataset using the SMOTE-Tomek Link method showed no significant improvement (*p* > 0.05) over the original dataset, suggesting that the current sample distribution of low lodging (LL) and high lodging (HL) plots is sufficient for robust classification. However, Bazarfkan et al. [19] identified that the balanced dry peas dataset obtained using the SMOTE-ENN method showed higher accuracy, precision, recall and F1-score than the original dataset. This was mainly due to the significantly large differences in the non-lodging, light-lodging and sever-lodging datasets that were not observed in the dry bean dataset between LL and HL.

**Figure 5:**
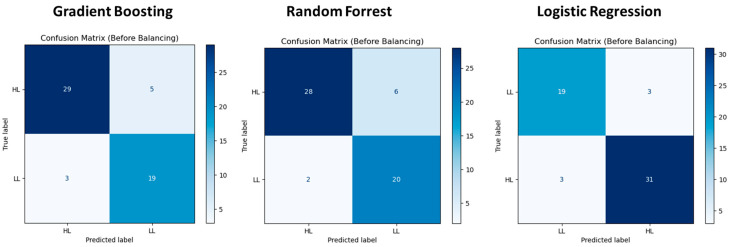
Confusion matrix for the best three models (test dataset for LL is 22 and HL is 34).

Figure 5 presents three confusion matrices illustrating the classification performance of each before dataset balancing. Each matrix visually compares predicted and actual class labels for HL and LL, with darker shades indicating higher classification counts. In the first confusion matrix (left), the Gradient Boosting correctly classifies 29 HL instances, misclassifying 5 as LL. For LL, 19 instances are correctly identified, while 3 are misclassified as HL. Random Forrest shows similar performance, with 28 correct HL predictions (6 misclassified) and 20 correct LL predictions (2 misclassified), indicating stable classification with minor variations.

Logistic Regression showed 19 correctly classified LL instances with 3 misclassified as HL, while HL has 31 correct predictions and 3 misclassified. Overall, Logistic Regression demonstrated strong classification accuracy, with the lowest misclassification variations among LL and HL that may be influenced by dataset imbalance.

**Figure 6:**
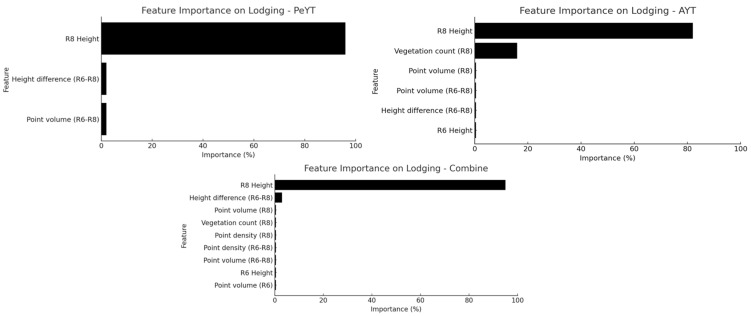
Feature dominance for lodging estimation (*p* < 0.05).

Figure 6 presents key features that significantly influenced lodging estimation across three different trial conditions: combined, PeYT and AYT. Plant height at R8 was consistently the most dominant feature across all three trials, emphasizing that plant height at the maturity stage is a strong predictor of lodging susceptibility. These results align with Bazrafkan et al. [19], who found that canopy height was a key predictor for dry pea lodging resistance, further validating the use of the LiDAR-derived structural features for lodging assessment.

In the combined trial, a wide range of features significantly contributed to lodging estimation. This aligns with previous findings in soybean, where Konno and Homma [23] observed that thicker, well-developed stems at the R6 stage improved structural integrity, reducing lodging risk at maturity. In PeYT trials, lodging scores were predominantly low (ratings of 2 and 3), supporting the hypothesis that taller, more structurally stable plants resist lodging more effectively. The inclusion of height difference between R6 and R8 suggests that changes in plant height over time are closely linked to lodging risk. In the PeYT trial, the number of significant features is slightly reduced compared to the combined trial, but height-related variables (R8 and R6) remain dominant. Additionally, point volume and vegetation count at the R8 stage continued to play an important role. This suggested that lodging estimation in PeYT relies primarily on height variations and canopy structure captured through the LiDAR-derived volume measurements.

Overall, the analysis reveals that height dynamics, vegetation structure and the LiDAR-derived point-based metrics play a crucial role in lodging estimation, with some variations across different trials.

### 3.3. Seed Yield Estimation

#### 3.3.1. LiDAR-Based Yield Estimation

For seed yield estimation using LiDAR, all the point cloud features for the polygon-based plots were considered. For the combined dataset, GBRT demonstrated the highest predictive performance, while RFR was termed to be the second-best among all the models (Table 4). However, there is no statistical significance (*p* > 0.05) in their metrics, suggesting both could be used to explore further improvement protocols.

Further analysis indicated that both GBRT and RFR exhibit better performance on the PeYT dataset than the AYT dataset, as evidenced by higher R^2^ values and lower RMSE and MAE values. The higher R^2^ for PeYT can be attributed to the stronger correlation between early flowering (R1) LiDAR height and yield (*p* = 0.0003) compared to AYT (*p* = 0.0111), reinforcing the importance of early flowering structural traits in predicting final yield.

#### 3.3.2. MSI-Based Yield Estimation

The average values of the NDVI for each polygon-based plot were computed to correlate with the ground-measured dry bean seed yield (kg/ha). For the combined dataset, RFR demonstrated the highest predictive performance, while GBRT was termed to be the second-best among all the models (Table 5). However, there is no statistical significance (*p* > 0.05) in their metrics, suggesting both could be used to explore further improvement protocols.

In addition, it was observed that AYT exhibited the highest R^2^ in comparison to PeYT, irrespective of the model type. This trend was the opposite for LiDAR data. This was due to their significance value with R6 NDVI for AYT, resulting in *p* = 1.576 × 10^−13^. However, the RMSE and MAE trend is similar in both imaging techniques, which could be due to the inclusion of sub-trials in AYT plots. In addition, feature analysis indicated that R6 NDVI affected combined, PeYT and AYT trials, respectively, with R1 NDVI impacting only combined and AYT trials. However, Wang et al. [6] showed that the R8 NDVI of AYT plots containing Yellow and Pinto bean trials were more promising to predict the dry bean yield but with a lower R^2^ of 0.14.

#### 3.3.3. Integrated LiDAR and MSI-Based Yield Estimation

**Figure 7:**
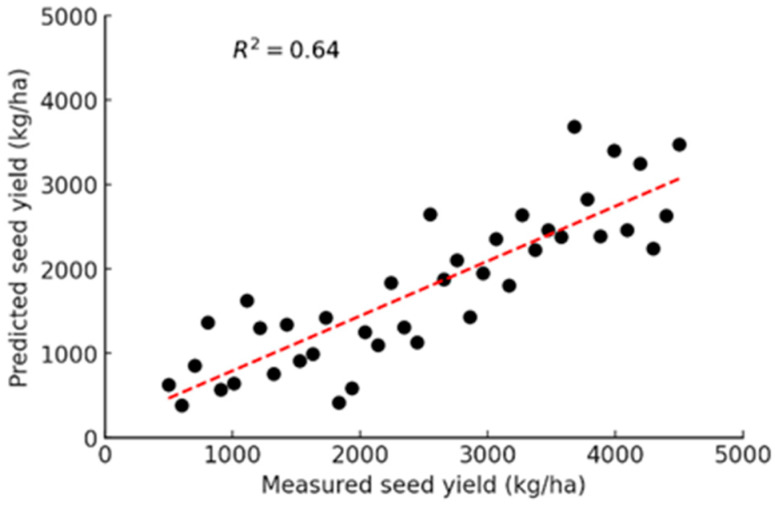
Correlation between predicted (GBRT model using combined trial) and measured seed yield.

Among the models, GBRT demonstrated the highest predictive performance in the combined dataset, indicating its ability to effectively capture complex relationships between LiDAR and MSI features (Table 6), followed by RFR. A visual representation of the correlation between measured seed yield with the predicted yield is shown in Figure 7. MLR also performed relatively well, suggesting that linear dependencies exist within the dataset, though nonlinear models captured more intricate patterns.

For the PeYT dataset, GBRT and RFR continued to perform well, with RFR achieving the highest R^2^, followed closely by GBRT. The lower RMSE and MAE values in PeYT (compared to AYT) suggested that models generalized better in this dataset. In contrast, the AYT dataset exhibited higher RMSE values, indicating greater prediction errors. The models struggled more with AYT, suggesting higher variability in the trials.

**Figure 8:**
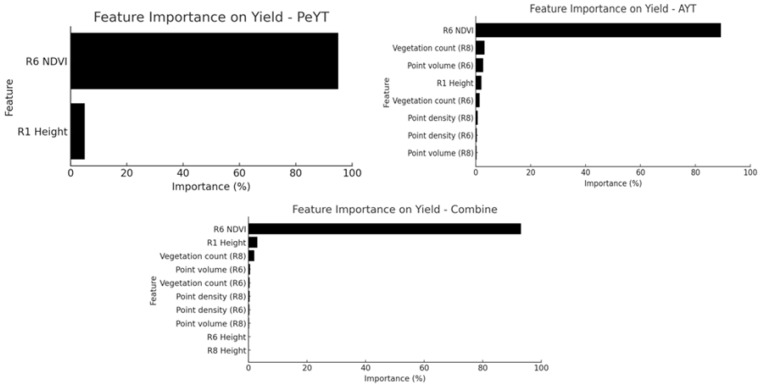
Feature dominance for seed yield estimation using LiDAR and MSI dataset (*p* < 0.05).

Figure 8 provides insight into feature dominance across datasets, revealing the reason for PeYT outperforming the AYT trial. The most significant predictor across all datasets was R6 NDVI, indicating that the multispectral sensor-based spectral vegetation index played a key role in yield estimation.

However, with AYT, the inclusion of additional point cloud variables suggests the impact of AYT-based sub-trials. This suggests that yield variation in AYT was primarily linked to the LiDAR-derived structural attributes rather than spectral reflectance differences alone, contributing to the higher RMSE and lower R^2^ values, as structural features alone may not fully capture plant physiological variations that impact yield [24]. Overall, the results indicate that superior performance is due to the availability of both LiDAR and MSI-based predictive features, allowing models to capture a broader range of factors influencing yield.

To enhance AYT predictions, future work should consider incorporating additional multispectral indices or feature engineering techniques to better represent plant physiological differences and reduce prediction errors.

While this study highlights the advantages of UAV-based LiDAR and MSI for estimating key dry bean traits, several limitations should be acknowledged. First, the experiments were conducted at a single geographic location and within a single growing season. Although the inclusion of multiple trials (PeYT and AYT) and AYT sub-trials provided internal variability, ongoing multi-year and multi-location studies are necessary to assess the broader applicability of the models across diverse environments. Second, although LiDAR delivers high spatial resolution and detailed canopy structure information, its high cost poses a challenge. Breeding programs must weigh the benefits of improved phenotyping accuracy against the financial investment required for LiDAR systems. Third, while the models developed in this study have proven effective for dry bean and show potential applicability to similar legume crops (as supported by prior research), extending them to crops with different canopy architectures, such as dense-cover or low-lying species and various cereal crops may require adjustments in data processing and feature extraction approaches.

## 4. Conclusions

This study highlights the effectiveness of UAV-based LiDAR and multispectral imaging for non-destructive, high-throughput phenotyping of dry bean breeding trials. In this study, a total of 15 crop features of LiDAR point cloud and three multispectral features were considered to estimate plant height (using canopy height), crop lodging and seed yield (using digital biomass). These features included vegetation count, point density, point volume, canopy height, NDVI and three maturity indices that were determined for R1, R6 and R8 crop growth stages, respectively. Regression and classification models were utilized and optimized based on the relevant evaluation matrices.

The LiDAR-derived canopy height detection exhibited strong correlations with plant height data, with the mid-pod filling stage (R6) showing the highest accuracy (R^2^ = 0.84). Crop lodging classification was best achieved using Gradient Boosting, Random Forest and Logistic Regression models, with plant height at the R8 stage being the most dominant feature for lodging resistance estimation with the classification accuracy of 0.80 for LL and HL, respectively. Seed yield prediction improved when combining LiDAR and MSI, with GBRT achieving the highest accuracy (R^2^ = 0.64), with the R6 stage NDVI feature being the most dominant.

The study confirms that UAV-based LiDAR and MSI offer a viable alternative to traditional phenotyping methods, enabling rapid and precise assessment of key agronomic traits. The integration of both imaging modalities significantly enhanced the accuracy of seed yield prediction, facilitating the development of high-yielding, resilient dry bean cultivars.

Seed yield estimation using MSI in this study was based on predefined spectral indices, specifically utilizing the NIR and Red bands to compute the NDVI for dry bean plots. While this approach provided useful insights, there is potential to improve model performance and robustness by incorporating all six spectral bands into machine learning models. Leveraging the full spectral range may allow for the development of crop-specific formulations that better capture the physiological characteristics of dry bean cultivars. To ensure consistency and broader applicability, future research should also emphasize multi-year validation and include trials across different dry bean varieties. Although the current study is based on a single-year dataset, ongoing multi-year and multi-variety trials are being conducted to validate and strengthen the generalizability of these findings. These efforts will be critical for optimizing model reliability and expanding the utility of LiDAR and MSI-based approaches in dry bean breeding programs.

## Figures and Tables

**Table 1:** Ground and UAV data collection dates.

Stages	Traits	UAV Dates	Ground Sampling Date (AYT/PeYT)
Mid-flowering (R1)	Height	22 July 2024	17 July 2024
Mid-pod filling (R6)	Height	13 August 2024	12 August 2024
Physiological maturity (R8)	Height	5 September 2024	4 September 2024
	Lodging		21 August to 14 September 2024
	Yield		25 September 2024 (Harvest day)

**Table 2:** Lodging scale classification scheme.

Trials	LL		HL	
	2	3	4	5
AYT	14	52	100	6
PeYT	5	39	50	10
Combine	19	91	150	16

**Table 3:** Evaluation matrices for the classification models (using original dataset and gray color indicates the best performing model).

Models	LL (2 and 3 Scales)				HL (4 and 5 Scales)			
	Accuracy	Precision	Recall	F1-Score	Accuracy	Precision	Recall	F1-Score
AB	0.71	0.68	0.69	0.69	0.73	0.71	0.70	0.71
GB	0.77	0.70	0.73	0.71	0.77	0.82	0.79	0.81
KNN	0.65	0.61	0.63	0.62	0.68	0.65	0.67	0.66
LGB	0.67	0.63	0.65	0.64	0.72	0.67	0.71	0.69
RF	0.75	0.67	0.73	0.70	0.75	0.81	0.76	0.79
SVM	0.62	0.58	0.61	0.59	0.68	0.62	0.65	0.63
XGBoost	0.71	0.69	0.70	0.69	0.75	0.71	0.73	0.72
LR	0.80	0.72	0.82	0.77	0.80	0.87	0.79	0.83

**Table 4:** Performance metrics of regression models for LiDAR-based yield estimation (gray color indicates the best performing model).

Model	Combined			PeYT			AYT		
	R^2^	RMSE	MAE	R^2^	RMSE	MAE	R^2^	RMSE	MAE
ANN (1024, 512) ^1^	0.26	979	677.2	0.27	572.4	541.3	0.18	1384.1	783.5
GBRT (learning rate 0.2) ^2^	0.45	883	687.6	0.41	436.4	408.8	0.26	1135.8	839.8
RF	0.33	941.4	681.7	0.51	469.1	415.6	0.15	1039.3	791.3
PLSR	0.12	1074.4	883.2	0.23	581.3	559.6	0.11	1263.6	892.4
MLR	0.24	993.1	749.8	0.31	629.4	602.1	0.14	1193.6	838.8

^1^ Denotes the optimized layer numbers; ^2^ denotes the optimized learning rate.

**Table 5:** Performance metrics of regression models for MSI-based yield estimation (grey color indicates the best performing model).

Model	Combine			PeYT			AYT		
	R^2^	RMSE	MAE	R^2^	RMSE	MAE	R^2^	RMSE	MAE
ANN (1024, 512) ^1^	0.25	912.2	786.1	0.27	628.4	537.5	0.31	944.2	748.2
GBRT (learning rate 0.2) ^2^	0.53	756.2	579.3	0.25	578.9	436.9	0.48	814.4	621.2
RFR	0.57	723.1	531	0.24	584.8	460.6	0.47	820.1	632.3
PLSR	0.31	916.7	740.2	0.29	853.3	693.5	0.34	932.4	745.2
MLR	0.40	850.1	680.7	0.36	735.4	534.4	0.42	842.4	735.9

^1^ Denotes the optimized layer numbers; ^2^ denotes the optimized learning rate.

**Table 6:** Performance metrics of regression models (grey color indicates the best performing model).

Model	Combine			PeYT			AYT		
	R^2^	RMSE	MAE	R^2^	RMSE	MAE	R^2^	RMSE	MAE
ANN (1024, 512) ^1^	0.26	867.3	739.7	0.28	684.4	539.3	0.29	1038.3	893.4
GBRT (learning rate 0.2) ^2^	0.64	687.2	521.6	0.41	435.5	391.8	0.49	935.6	709.4
RFR	0.52	760.4	552.3	0.48	483	418	0.47	820.2	616.2
PLSR	0.25	955.4	763.3	0.23	738.4	639.4	0.26	983.4	832.3
MLR	0.5	804.1	640.1	0.43	784.5	645.2	0.46	842.6	742.7

^1^ Denotes the optimized layer numbers; ^2^ denotes the optimized learning rate.

## Data Availability

Data are contained within the article.

## Abbreviations

The following abbreviations are used in this manuscript:

LiDAR	Light Detection and Ranging
RGB	Red, Green and Blue
UAV	Unmanned Aerial Vehicles
r	Pearson correlation coefficient
R^2^	Coefficient of Determination
t	Tonnes
ha	Hacter
MSI	Multispectral image
DB	Digital biomass
AYT	Advanced Yield Trial
PeYT	Performance Yield Trial
YL	Yellow bean
PT	Pinto bean
GN	Great Northern bean
PM	Physiological Maturity
kg	Kilogram
N	North
W	West
CH	Canopy height
CL	Crop Lodging
GCP	Ground Control Point
ROI	Region of Interest
LAS	LASer
LL	Low Lodging
HL	High Lodging
ML	Machine Learning
AB	Adaptive Boosting
GB	Gradient Boosting
KNN	K-Nearest Neighbors
LGB	Light Gradient Boosting
RF	Random Forrest
SVM	Support Vector Machine
XGBoost	Extreme Gradient Boosting
LR	Logistic Regression
SMOTE-ENN	Synthetic Minority Oversampling-Edited Nearest Neighbor
ADASYN	Adaptive Synthetic
NDVI	Normalized Difference Vegetation Index
ANN	Artificial Neural Network
GBRT	Gradient Boosting Regression Trees
PLSR	Partial Least Square Regression
MLR	Multiple Linear Regression
RMSE	Root Mean Square Error
MAE	Mean Absolute Error
NIR	Near Infrared
